# Chrononutrition Behaviors and BMI Change During the Transition to University Life in Young Adults: A Prospective Study

**DOI:** 10.3390/nu18121975

**Published:** 2026-06-18

**Authors:** Călin Muntean, Teodora Piroș, Ruxandra-Cristina Marin, Lavinia Cristina Moleriu, Raluca Lupușoru, Anca Mihaela Dicu, Sebastian Ștefănigă, Radu Dumitru Moleriu

**Affiliations:** 1 Department III Functional Science, Discipline of Medical Informatics and Biostatistics, “Victor Babes” University of Medicine and Pharmacy, 300041 Timisoara, Romania; cmuntean@umft.ro (C.M.); teodora.piros@student.umft.ro (T.P.); raluca.lupusoru@umft.ro (R.L.); radu.moleriu@umft.ro (R.D.M.); 2 Discipline of Pharmacology, Clinical Pharmacology and Pharmacotherapy, “Carol Davila” University of Medicine and Pharmacy, 050474 Bucharest, Romania; 3 Doctoral School of Biological and Biomedical Sciences, University of Oradea, 410087 Oradea, Romania; 4 Center for Modeling Biological Systems and Data Analysis, “Victor Babes” University of Medicine and Pharmacy, 300041 Timisoara, Romania; 5 Gastroenterology and Hepatology Clinic, County Emergency Hospital “Pius Brinzeu”, 300723 Timisoara, Romania; 6 Faculty of Food Engineering, Turism and Environmental Protection, “Aurel Vlaicu” University of Arad, 310330 Arad, Romania; anca.dicu@uav.ro; 7 Faculty of Computer Science, West University of Timisoara, 300223 Timișoara, Romania; sebastian.stefaniga@e-uvt.ro

**Keywords:** chrononutrition, meal timing, BMI trajectory, young adults, university students, prospective study, obesity prevention

## Abstract

**Background**: The transition to university life is characterized by substantial changes in eating behaviors, sleep–wake organization, and lifestyle patterns. Chrononutrition-related behaviors, including meal timing and caloric distribution across the day, may influence metabolic health independently of dietary quantity, yet prospective evidence in university students remains limited. This study aimed to evaluate BMI trajectories during the first academic year and to identify chrononutrition-related behaviors associated with BMI change and clinically significant weight gain in young adults. **Methods**: This prospective observational study included 921 university students aged 18–24 years. BMI was assessed at university entry and after one academic year, and BMI change (Diff_BMI) was calculated. Chrononutrition-related variables included meal frequency, identity of the main meal, breakfast habits, and late eating. Multivariable linear and logistic regression models were used to evaluate independent associations between chrononutrition-related behaviors, BMI change, and clinically significant weight gain (Diff_BMI > +1 kg/m^2^). **Results**: Mean Diff_BMI was −0.45 ± 1.07 kg/m^2^ (95% CI −0.52 to −0.38; *p* < 0.001). Overall, 48.2% of participants showed BMI reduction, 38.7% remained weight-stable, and 13.1% experienced BMI increases; clinically significant weight gain occurred in 7.2% of the cohort. In multivariable analysis, having dinner as the main meal (vs. lunch) was independently associated with greater BMI gain (β = +0.22 kg/m^2^; *p* = 0.004). Male sex was associated with lower BMI gain and lower odds of clinically significant weight increase. Breakfast skipping showed an inverse association with BMI change, whereas meal frequency and late eating were not independently associated with BMI trajectory after adjustment. **Conclusions**: The temporal distribution of caloric intake, particularly late eating patterns and shifting the principal meal toward later hours of the day, appears more strongly associated with BMI trajectory during the transition to university life than meal frequency alone. These findings support the relevance of chrononutrition-oriented strategies targeting meal timing and circadian eating behaviors in university students.

## 1. Introduction

Overweight and obesity remain major public-health challenges among young adults, a life stage during which dietary, sleep, and lifestyle habits become increasingly consolidated [1]. The transition from adolescence to independent living is frequently accompanied by changes in eating behaviors, physical activity, sleep patterns, and body-weight regulation, making emerging adulthood an important period for obesity prevention [2,3,4,5]. University students are particularly vulnerable because academic stress, irregular schedules, altered sleep–wake patterns, and increased exposure to convenience foods may adversely affect metabolic health [6,7,8,9].

The university environment may also contribute to chronobiological disruption. Irregular schedules, evening studying, and social activities may promote social jetlag and reduced daytime activity, while delayed eating patterns and reduced dietary regularity may further influence metabolic regulation [10,11]. These behavioral changes have been increasingly recognized as potential contributors to unfavorable weight-related outcomes during the university transition.

Although the “Freshman Fifteen” concept has traditionally emphasized weight gain during the first year of university, contemporary studies suggest that BMI trajectories are heterogeneous rather than uniformly upward. First-year university students show substantial interindividual variability in body-weight change, supporting the idea that some students gain weight, whereas others remain stable or lose weight [12]. This heterogeneity may be partly explained by the transition from structured family-based eating environments to more autonomous dietary behaviors during university life. Evidence suggests that the transition from high school to university represents a vulnerable period for the development of unhealthy dietary and lifestyle patterns that may adversely affect metabolic health [13].

Beyond the traditional paradigm of energy balance, chrononutrition emphasizes that the timing, regularity, and circadian alignment of food intake may influence metabolic health independently of total caloric intake [14]. Food intake acts not only as a source of energy but also as a biological timing cue capable of synchronizing peripheral metabolic clocks involved in glucose metabolism, lipid handling, appetite regulation, and energy expenditure [15,16]. Consequently, circadian misalignment between eating schedules and endogenous biological rhythms has been associated with impaired metabolic regulation, increased adiposity, and adverse cardiometabolic outcomes [17,18,19]. Among young adults, chrononutrition-related behaviors such as breakfast skipping, delayed eating patterns, and shifting a greater proportion of daily energy intake toward later hours of the day have attracted growing interest because they may contribute to obesity-related outcomes and broader metabolic vulnerability [20].

Short sleep duration has also been linked with skipping major meals among university students, suggesting that eating behaviors may be embedded within broader circadian and sleep-related disruption [21]. Late or evening-weighted eating may be metabolically unfavorable because meal-timing strategies influence anthropometric and metabolic outcomes, although effects may depend on the specific timing pattern, energy distribution, and population studied [22].

Chrononutrition-related behaviors rarely occur in isolation. Breakfast skipping, late eating, irregular meal timing, shortened sleep duration, sedentary behavior, and stress-related eating frequently cluster together in young-adult populations, complicating the interpretation of individual dietary behaviors when examined separately. Therefore, multivariable approaches evaluating several chrononutrition dimensions may provide a more ecologically valid representation of real-world eating patterns during the university transition.

Despite growing interest in chrononutrition, evidence in students undergoing the transition to university remains fragmented. Many studies focus on isolated behaviors such as breakfast skipping, sleep, or physical activity, whereas fewer simultaneously examine meal frequency, breakfast habits, late eating, and the position of the principal daily meal within the same analytical framework. This gap is important because meal frequency alone may not capture the metabolic relevance of whether the largest caloric load is consumed earlier or later in the day.

Therefore, this prospective study aimed to evaluate BMI changes over one academic year in young adults aged 18–24 years and to explore the chrononutrition-related behaviors associated with BMI trajectory during the transition to university life. Specifically, the study examined daily meal frequency, habitual breakfast consumption, breakfast composition, main-meal distribution throughout the day, and late eating in relation to BMI change and clinically significant weight gain. By evaluating several dimensions of eating timing and meal organization within the same analytical framework, the study sought to provide a more comprehensive understanding of how chrononutrition-related behaviors may influence weight-related outcomes in university students. It was hypothesized that meal-timing behaviors, particularly late eating patterns and shifting the principal meal toward later hours of the day, would be associated with greater BMI gain independently of demographic characteristics. Because chrononutrition-related behaviors were assessed only at follow-up, the observed associations should be interpreted as concurrent correlates or behavioral markers associated with BMI trajectory rather than temporally established causal or predictive determinants of BMI change during the study period.

## 2. Materials and Methods

### 2.1. Study Design, Setting, and Participants

This single-center prospective observational study was conducted among young adults enrolled at a public university over one consecutive academic year. Two assessment points were considered: baseline (T0), corresponding to university entry, and follow-up (T1), corresponding to the end of the first academic year. The target population consisted of students enrolled in the Romanian-language General Medicine program at the “Victor Babeș” University of Medicine and Pharmacy, Timișoara, across all six years of study during the 2025–2026 academic year. Based on the official admission quota of 459 students per academic year, the eligible target population was estimated at approximately 2754 students. A total of 967 students participated in the baseline assessment, and 921 students completed both assessments and met all eligibility criteria for inclusion in the final analysis, corresponding to approximately 33.4% of the eligible student population. At both time points, anthropometric data were obtained through self-reported height and weight collected via online questionnaires rather than direct clinical examination. Self-reported anthropometric assessment was selected because of the large-scale prospective design and the non-interventional university setting, where direct standardized measurements were not logistically feasible. The same self-report methodology was applied consistently at both assessment points to improve internal comparability of BMI trajectory estimates. Participants received brief instructions regarding height and weight reporting within the questionnaire, and the university-student population included individuals reasonably familiar with basic anthropometric and health-related concepts. Self-reported height and weight are also commonly used in epidemiological studies involving young-adult and university populations. BMI values calculated from T0 data were designated as BMI_previous, whereas those derived from T1 were defined as BMI_actual.

Eligible participants included students aged 18–24 years who reported no chronic conditions or pharmacological treatments known to influence body weight and who were not pregnant or lactating. Individuals with incomplete anthropometric self-reports or missing chrononutrition questionnaire data were excluded from the final analysis.

967 students initially responded to the baseline assessment. Of these, 46 participants were excluded because of incomplete follow-up participation, had missing height or weight data at either assessment point, or incomplete chrononutrition questionnaire data. Consequently, a total of 921 students completed both assessments and were included in the final study population.

### 2.2. Data Collection, Variables, and Measurements

Data were collected using a structured self-administered online questionnaire created using Google Forms (Google LLC, Mountain View, CA, USA) distributed through institutional electronic mailing systems and social media platforms. The questionnaire (Supplementary File S1: English version of the questionnaire) included an Introductory Section describing the study objectives, the voluntary nature of participation, and the confidentiality measures applied to data handling and analysis. The present analysis specifically focused on selected chrononutrition-related variables relevant to meal timing and temporal eating behaviors, whereas additional lifestyle- and nutrition-related domains collected within the broader questionnaire framework were evaluated separately in parallel analyses.

Chrononutrition-related variables were assessed at T1 using a structured questionnaire developed using chrononutrition-related questions previously employed in studies involving adult and university student populations. The questionnaire was designed to evaluate temporal eating behaviors and meal-timing patterns.

The assessed variables included daily meal frequency (2–3 vs. 4–5 meals/day), identification of the self-perceived main meal of the day (breakfast, lunch, or dinner), defined according to the participant’s subjective perception rather than a standardized caloric or portion-based definition, daily breakfast consumption assessed through the question “Do you eat breakfast every morning?” (“yes/no”), inclusion of a substantive main dish at breakfast (yes/no), and late eating (before 18:00, 19:00–20:00, 21:00–22:00, or after 22:00). Throughout the manuscript, this variable is referred to as ‘late eating’ when discussing meal timing patterns and their associations with BMI-related outcomes. Participants answering “no” to the breakfast question were classified as breakfast skippers, whereas those answering “yes” were categorized as daily breakfast consumers for the purposes of the analyses.

The primary outcome variable, Diff_BMI, was defined as the difference between BMI measured at follow-up (BMI_actual) and BMI measured at baseline (BMI_previous), expressed in kg/m^2^. Clinically significant weight gain was defined a priori as Diff_BMI > +1 kg/m^2^ to distinguish meaningful anthropometric changes from minor physiological variation.

### 2.3. Statistical Analysis

All statistical analyses were performed using JASP version 0.18.3 (JASP Team, Amsterdam, The Netherlands), using R version 4.3.0 (R Foundation for Statistical Computing, Vienna, Austria) as the computational backend.Continuous variables were assessed for distributional normality using graphical and statistical methods. Data are presented as mean ± standard deviation (SD) and median with interquartile range (IQR) for continuous variables, and as frequencies with percentages for categorical variables. Two-tailed statistical significance was set at α = 0.05.

Comparisons between baseline and follow-up BMI values were performed using paired parametric and non-parametric tests. Between-group comparisons used independent-samples tests or one-way analyses according to variable distribution and number of groups. Associations between categorical variables were assessed using chi-square tests, while correlations between chrononutrition-related variables and Diff_BMI were evaluated using Pearson and Spearman coefficients.

The association between late eating and Diff_BMI was additionally explored using linear regression and analysis of covariance (ANCOVA) adjusted for sex and age. Trend analyses across ordered late eating categories were evaluated using Jonckheere–Terpstra and ordinal linear-trend regression analyses.

Multivariable linear regression analyses were conducted using Diff_BMI as the dependent variable, whereas multivariable logistic regression analyses evaluated clinically significant weight gain (Diff_BMI > +1 kg/m^2^). Variables entered into the models included meal frequency, breakfast skipping, late eating, main meal identity, sex, and age stratum. Model assumptions, residual distribution, homoscedasticity, and multicollinearity were evaluated using standard diagnostic procedures and variance inflation factors (VIFs). Results are reported as regression coefficients (β), adjusted odds ratios (aORs), and 95% confidence intervals (CIs).

Pre-specified sensitivity analyses included adjustment for baseline BMI, sex-stratified regression analyses, interaction testing between sex and chrononutrition-related variables, effect-size estimation using Cohen’s d, and post hoc statistical power assessment.

The primary analyses evaluating dinner predominance and late eating in relation to BMI trajectory were prespecified. Additional subgroup, interaction, and sensitivity analyses were considered exploratory. Formal correction for multiple comparisons was not applied because of the mixed hypothesis-driven and exploratory nature of the analyses and the risk of excessive type II error inflation associated with highly conservative adjustment strategies.

### 2.4. Ethical Considerations

Participation in the study was voluntary, and all participants were informed about the study objectives, procedures, and intended use of the collected data prior to enrollment.

The study protocol was approved by the Ethics Committee of the “Victor Babeș” University of Medicine and Pharmacy Timișoara (approval no. 95/4 October 2021) and conducted in accordance with the Declaration of Helsinki and the principles of good biomedical research practice. Written informed consent was obtained from all participants prior to study inclusion.

## 3. Results

### 3.1. Characteristics of the Study Population

The analytic sample comprised 921 young adults (558 women, 60.6%; 363 men, 39.4%); 443 (48.1%) were aged 18–20 years and 478 (51.9%) were aged 21–24 years. The Shapiro–Wilk statistic indicated departures from normality for BMI_previous (W = 0.981, *p* < 0.001), BMI_actual (W = 0.972, *p* < 0.001), and Diff_BMI (W = 0.972, *p* < 0.001); the Kolmogorov–Smirnov test corroborated these findings for BMI_actual (D = 0.093, *p* < 0.001) and Diff_BMI (D = 0.071, *p* < 0.001), whereas BMI_previous approached normality (D = 0.039, *p* = 0.128). Because several variables demonstrated deviations from Gaussian distribution, non-parametric analyses were systematically performed in parallel to confirm the robustness of the findings.

BMI category distribution also changed between assessment points. At baseline (T0), 72 participants (7.8%) were classified as underweight, 531 (57.7%) had normal weight, 262 (28.4%) were overweight, and 56 (6.1%) had obesity. At follow-up (T1), the distribution shifted toward the normal-weight category, with 59 participants (6.4%) classified as underweight, 640 (69.5%) as normal weight, 179 (19.4%) as overweight, and 43 (4.7%) as having obesity. Notably, 107 participants who were overweight at baseline transitioned to normal weight at follow-up. Mean Diff_BMI was positive among participants who were underweight at baseline (+0.57 kg/m^2^) and negative among those who were overweight (−0.69 kg/m^2^) or obese (−0.73 kg/m^2^) at baseline. These descriptive patterns are consistent with the modest overall reduction in mean BMI observed at the cohort level.

Accordingly, both parametric and non-parametric tests are reported. Sex–chrononutrition associations were significant for main-meal identity (χ^2^ = 14.82, *p* < 0.001) and late eating (χ^2^ = 8.78, *p* = 0.032), but not for total meals per day, daily breakfast consumption, breakfast main dish, or age stratum (all *p* ≥ 0.17). Baseline characteristics are summarized in Table 1.

### 3.2. Changes in BMI During One Year of University

**Figure 1:**
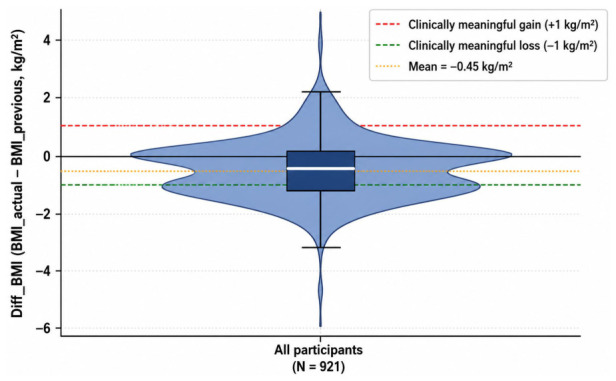
Distribution of Diff_BMI (BMI_actual − BMI_previous, kg/m^2^) after one academic year. The central marker represents the median, the box indicates the interquartile range (IQR), and dashed lines indicate ±1 kg/m^2^.

Across the cohort, BMI decreased significantly between T0 and T1 (paired t = −12.78, *p* < 0.001; Wilcoxon signed-rank W = 108,110.5, *p* < 0.001). Despite the overall reduction in mean BMI at cohort level, substantial interindividual variability in BMI trajectory was observed. Mean Diff_BMI was −0.45 ± 1.07 kg/m^2^ (95% CI −0.52 to −0.38; one-sample t vs. 0: t = −12.78, *p* < 0.001), with a wide individual range (−5.78 to +4.83 kg/m^2^). When categorized using a ±0.5 kg/m^2^ threshold, 444 participants (48.2%) exhibited a BMI decrease, 356 (38.7%) remained stable, and 121 (13.1%) showed a BMI increase. A clinically significant weight gain (Diff_BMI > +1 kg/m^2^) was observed in 66 participants (7.2%). These findings indicate that although average BMI tended to decrease during the first university year, a clinically relevant subgroup experienced meaningful weight gain. The patterns of weight change are detailed in Table 2 and the full distribution of Diff_BMI is illustrated in Figure 1.

### 3.3. Meal Frequency and BMI Change

**Figure 2:**
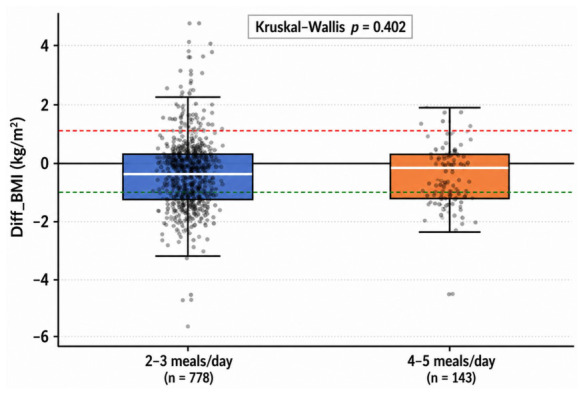
Distribution of Diff_BMI according to daily meal frequency. Boxes represent the interquartile range (IQR), horizontal lines indicate medians, and whiskers extend to 1.5 × IQR.

Participants reporting 2–3 meals per day (*n* = 778; 84.5%) and those reporting 4–5 meals per day (*n* = 143; 15.5%) did not differ in BMI trajectory: Diff_BMI was −0.46 ± 1.08 kg/m^2^ (median −0.39, IQR −1.21 to 0.18) versus −0.41 ± 1.00 kg/m^2^ (median −0.18, IQR −1.16 to 0.23), respectively. Neither one-way ANOVA (F = 0.24, *p* = 0.624) nor Kruskal–Wallis (H = 0.70, *p* = 0.402) detected a significant difference, and the Bonferroni-corrected pairwise comparison confirmed the null finding (*p* = 0.606). Pearson and Spearman correlations between meal frequency and Diff_BMI were negligible (r = 0.016, *p* = 0.624; ρ = 0.028, *p* = 0.402). Overall, effect size estimates consistently supported the absence of a clinically meaningful relationship between total daily meal frequency and BMI change during the first university year. Stratified estimates are presented in Table 3 and visualized in Figure 2.

**Figure 3:**
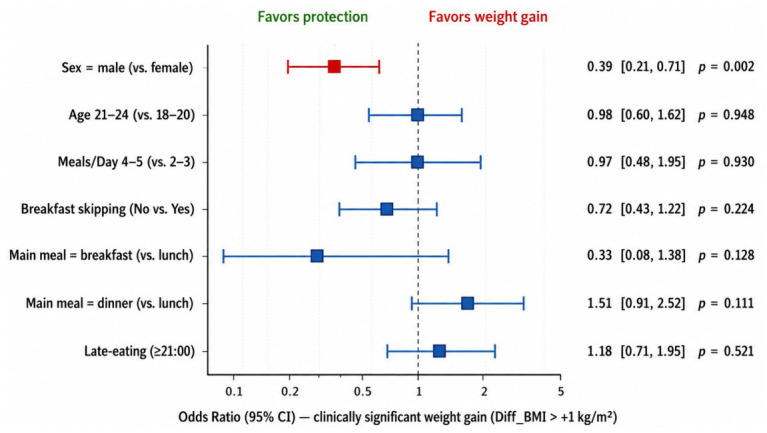
Univariable odds ratios (ORs) and 95% confidence intervals (CIs) for clinically significant weight gain (Diff_BMI > +1 kg/m^2^) according to chrononutrition-related and demographic variables. The dashed line indicates OR = 1.

Daily breakfast consumers (*n* = 274; 29.8%) had a mean Diff_BMI of −0.32 ± 0.98 kg/m^2^ (median −0.16), whereas breakfast skippers (*n* = 647; 70.2%) showed a mean Diff_BMI of −0.50 ± 1.10 kg/m^2^ (median −0.50). The two groups differed significantly with respect to Diff_BMI (Welch t = 2.50, *p* = 0.013; Mann–Whitney U = 98,598.0, *p* = 0.007), but not in BMI_actual (23.33 ± 4.01 vs. 23.25 ± 3.78 kg/m^2^; Welch t = 0.30, *p* = 0.767). Breakfast skipping was associated with a greater overall reduction in BMI during the observation period, although the magnitude of the association was modest. Consistent with this pattern, the univariable odds ratio for clinically significant weight gain associated with breakfast skipping was 0.72 (95% CI 0.43–1.22; *p* = 0.224), indicating no statistically significant increase in risk. Detailed comparisons are reported in Table 4, and the univariable forest plot of odds ratios is presented in Figure 3.

### 3.4. Late Eating and BMI Variation

Diff_BMI did not differ significantly across the four categories of late eating (one-way ANOVA F = 0.58, *p* = 0.627; Kruskal–Wallis H = 2.31, *p* = 0.510). Similarly, no dose–response relationship between progressively late eating and BMI trajectory was identified. Pearson and Spearman correlations of the ordinal late eating score with Diff_BMI were negligible (r = 0.012, *p* = 0.712; ρ = 0.014, *p* = 0.680). Linear regression with “before 18:00” as the reference yielded non-significant coefficients for all later categories, and ANCOVA adjusted for sex and age produced equivalent results (all *p* ≥ 0.27). Bonferroni-corrected pairwise comparisons did not reveal any contrast with *p* < 0.05. Details are reported in Table 5.

To formally assess whether progressively late eating was associated with BMI change, a non-parametric Jonckheere–Terpstra trend test was performed across the four ordered late eating categories. The test confirmed the absence of a monotonic dose–response relationship (J = 144,380.5; z = 0.41; two-sided *p* = 0.685). An ordinal linear-trend regression yielded a comparable null estimate (β = 0.015 kg/m^2^ per category; 95% CI −0.068 to 0.099; *p* = 0.711), which remained non-significant after adjustment for sex and age stratum (β = 0.015; *p* = 0.721). These convergent results indicate that, in the present cohort, neither categorical nor ordinal modeling supported a graded association between progressively late eating and BMI trajectory.

### 3.5. Multivariable Correlates of BMI Trajectory and Clinically Significant Weight Gain

The multiple linear regression model with Diff_BMI as outcome was statistically significant overall (F(7, 913) = 3.47, *p* = 0.001; adjusted R^2^ = 0.018). Although the model reached statistical significance overall, its explanatory performance was limited (adjusted R^2^ = 0.018), indicating that the included variables accounted for only a small proportion of the variability in BMI trajectory. Dinner as the main meal of the day (versus lunch) was an independent positive correlate of BMI change (β = 0.22 kg/m^2^; 95% CI 0.07 to 0.37; *p* = 0.004), while female sex was independently associated with greater BMI gain (β for male sex = −0.15; 95% CI −0.29 to −0.01; *p* = 0.040). Breakfast skipping retained a significant but inversely directed association with Diff_BMI (β = −0.19; 95% CI −0.35 to −0.04; *p* = 0.013). Late eating, total meal frequency, age stratum, and breakfast-as-main-meal did not reach statistical significance in the adjusted model. In the logistic regression analysis for clinically significant weight gain, male sex emerged as a strong independent protective factor (aOR 0.36; 95% CI 0.19–0.66; *p* < 0.001). Overall, the multivariable findings suggest that the temporal distribution of caloric intake may be more strongly associated with BMI trajectory during the transition to university life than meal frequency alone. The fully adjusted models are detailed in Table 6.

### 3.6. Sensitivity and Sex-Stratified Analyses

**Figure 4:**
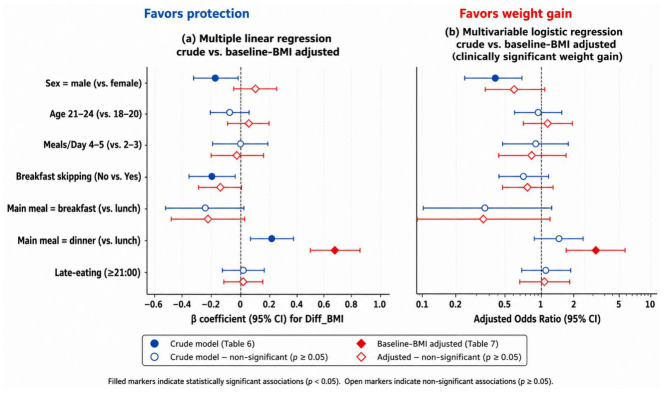
Regression coefficients and odds ratios from crude and baseline-BMI-adjusted multivariable models. Filled markers indicate *p* < 0.05.

To account for the potential influence of baseline adiposity on subsequent BMI trajectory and to evaluate the robustness of the primary findings, the main multivariable models were re-estimated with baseline BMI (BMI_previous) included as an additional covariate. After this adjustment, the explanatory performance of the linear model increased substantially (adjusted R^2^ increasing from 0.018 to 0.126). Baseline BMI was inversely associated with subsequent BMI change (β = −0.111 per kg/m^2^; 95% CI −0.131 to −0.090; *p* < 0.001), indicating a substantial influence of initial adiposity on subsequent BMI trajectory. Importantly, the independent association between having dinner as the main meal of the day and greater BMI gain became substantially stronger after adjustment for baseline BMI (β = 0.67; 95% CI 0.51 to 0.83; *p* < 0.001), while the corresponding adjusted odds ratio for clinically significant weight gain increased to 2.91 (95% CI 1.61–5.27; *p* < 0.001). Conversely, the previously significant associations of male sex and breakfast skipping with BMI change attenuated below the conventional significance threshold (male sex: β = 0.10, *p* = 0.154; breakfast skipping: β = −0.13, *p* = 0.068), suggesting that these associations were partly influenced by baseline differences in BMI. The complete baseline-BMI-adjusted models are presented in Table 7, while the comparison between crude and baseline-BMI-adjusted coefficients is illustrated in Figure 4.

Exploratory stratified analyses according to baseline BMI category (underweight/normal weight vs. overweight/obesity) suggested that the positive association between having dinner as the main meal of the day and greater BMI gain was more pronounced among participants who were overweight or obese at baseline (β = 0.31, *p* = 0.012) compared with those with underweight or normal weight at baseline (β = 0.12, *p* = 0.21) (Table 8). Although the categorical interaction term did not reach conventional statistical significance, formal interaction testing using continuous baseline BMI demonstrated a significant positive interaction between baseline BMI and dinner-as-main-meal (β for interaction = 0.080 per kg/m^2^, *p* < 0.001). Similarly, when baseline BMI was dichotomized at 25 kg/m^2^, the association between dinner predominance and Diff_BMI was significantly stronger among participants who were overweight or obese at baseline (additional β = 0.38 kg/m^2^, *p* = 0.024).

**Figure 5:**
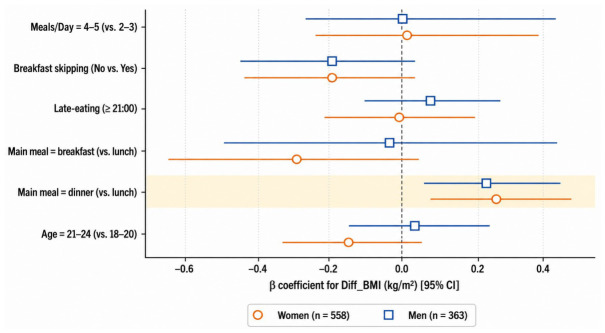
β coefficients and 95% confidence intervals from sex-stratified regression models for Diff_BMI. Orange circles represent women and blue squares represent men. Filled markers indicate *p* < 0.05; the dashed line denotes β = 0. The shaded yellow area is used to visually emphasize the “Main meal = dinner (vs. lunch)” predictor.

To explore whether the relationship between chrononutrition-related behaviors and BMI trajectory differed according to sex, the multiple linear regression model was refitted separately in women and men. The positive independent association between having dinner as the main meal of the day and BMI change was observed in both sexes and was highly comparable in magnitude (women: β = 0.23, *p* = 0.037; men: β = 0.22, *p* = 0.031), indicating that the association between evening caloric predominance and BMI gain was not sex-specific. Breakfast skipping showed a directionally consistent inverse association in both strata (women: β = −0.19; men: β = −0.19), although neither association reached statistical significance after stratification (both *p* = 0.08). Although the overall regression model in men did not reach conventional statistical significance, the consistency of the regression coefficients across sexes supports the stability of the identified chrononutrition patterns. The detailed sex-stratified regression models are presented in Table 9, and the corresponding regression coefficients are visually summarized in Figure 5.

**Figure 6:**
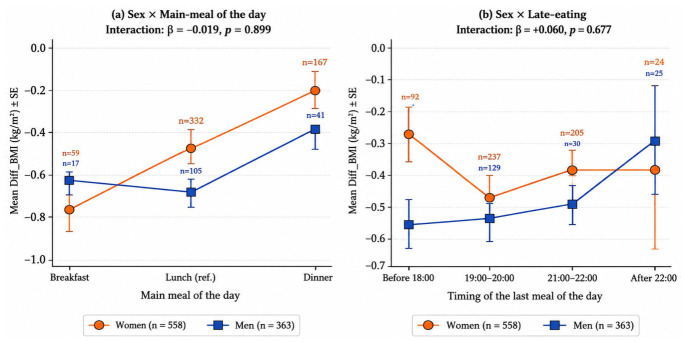
Sex × chrononutrition interaction plots for Diff_BMI. (**a**) Mean Diff_BMI according to main-meal category in women (orange circles) and men (blue squares). (**b**) Mean Diff_BMI according to timing of the last meal of the day in women (orange circles) and men (blue squares). Error bars represent standard errors (SE). Numbers above each data point indicate the number of participants within each category. The interaction coefficients (β) and corresponding p-values shown above each panel are derived from the multivariable interaction models reported in Table 10.

To formally evaluate potential effect modification by sex, multiplicative interaction terms were introduced into the multivariable linear regression model. Neither the interaction between sex and dinner-as-main-meal (β = −0.019; *p* = 0.899) nor the interaction between sex and late eating (β = 0.060; *p* = 0.677) reached statistical significance. The joint model including both interaction terms remained statistically significant overall (adjusted R^2^ = 0.017; *p* = 0.004) but provided no statistical evidence that sex modified the associations between chrononutrition-related behaviors and BMI trajectory. The interaction analyses are detailed in Table 10, while the corresponding interaction plots are shown in Figure 6.

Effect-size analyses provided additional context for the bivariate findings. Cohen’s d for the comparison of BMI change between participants reporting 2–3 versus 4–5 meals/day was negligible (d = −0.045; 95% CI −0.223 to 0.134), whereas the corresponding effect size for daily breakfast consumers versus breakfast skippers was small (d = 0.17; 95% CI 0.03 to 0.31). These findings reinforce that, despite statistical significance in selected comparisons, the observed effect magnitudes remained limited. A post hoc power analysis indicated that the present study design (*n* = 921; α = 0.05; seven independent associates) provided ≥90% statistical power to detect a small overall effect size (Cohen’s f^2^ = 0.02) in the multiple linear regression model, supporting adequate statistical sensitivity for detecting small associations in the multivariable analyses.

## 4. Discussion

In this prospective cohort of 921 university students followed across the first academic year, BMI trajectory showed considerable heterogeneity despite a modest overall decrease in mean BMI. Nearly half of the participants experienced BMI reduction, more than one third remained stable, and a smaller but clinically relevant subgroup showed BMI gain, including 7.2% who met the predefined threshold for clinically significant weight gain. The main finding was that having dinner as the principal meal of the day was the most consistent chrononutrition-related correlate of BMI gain, whereas total meal frequency and absolute late eating were not independently associated with BMI trajectory. This association became stronger after adjustment for baseline BMI, suggesting that the temporal distribution of caloric intake may be more informative than simple eating frequency when evaluating weight-related changes during the transition to university life. These findings support the relevance of chrononutrition as a behavioral framework for understanding behavioral patterns associated with BMI trajectory and metabolic vulnerability in young adults during emerging adulthood.

The overall decrease in BMI observed in our cohort contrasts with the traditional “Freshman 15” narrative, which assumes generalized weight gain during the first year of university. However, recent prospective evidence suggests that weight trajectories among university students are heterogeneous and may depend on baseline adiposity, lifestyle adaptation, eating behavior, and physical activity patterns rather than following a uniform direction [12]. This variability likely reflects the transition from structured family-based environments to more autonomous lifestyles characterized by changes in sleep schedules, meal timing, stress exposure, food affordability, and physical activity [13]. Barriers such as academic stress, time constraints, irregular schedules, and limited food-management skills have also been identified as important determinants of unhealthy eating behaviors among university students [23]. Studies in university populations have further demonstrated that irregular meal timing, delayed sleep schedules, social jetlag, and last-eating behaviors are highly prevalent during emerging adulthood and may contribute to obesity-related metabolic vulnerability [24,25]. The coexistence of both BMI reduction and clinically significant BMI gain within the same cohort therefore supports the concept that university transition represents a metabolically vulnerable but behaviorally heterogeneous period rather than a universally obesogenic exposure.

The finding that dinner as the main meal was independently associated with BMI gain is highly consistent with the central premise of chrononutrition: the metabolic impact of food intake depends not only on quantity and quality, but also on timing and distribution across the day. Recent reviews emphasize that later energy intake and late eating-weighted caloric distribution are associated with increased adiposity and poorer metabolic outcomes, particularly when food intake is misaligned with circadian metabolic rhythms [26]. Chrononutrition literature has additionally highlighted the role of circadian misalignment, late chronotype, and irregular eating timing as contributors to obesity risk, metabolic dysregulation, and adverse cardiometabolic profiles, particularly among young adults exposed to irregular social and academic schedules [27,28].

Our findings further suggest that, in young adults undergoing the transition to university life, the identity of the principal meal may be more relevant than the number of eating occasions. Similar observations have been reported in chrononutrition research, where greater evening energy intake and lower morning energy intake were associated with increased adiposity and poorer metabolic outcomes independently of total caloric intake [26]. Experimental evidence also demonstrates that later caloric intake may increase hunger, reduce energy expenditure, and induce unfavorable alterations in adipose-tissue metabolic pathways [18]. Evidence further suggests that the circadian distribution of energy intake may play a more important role in body-weight regulation than meal frequency itself, particularly in the context of late eating patterns and shifting a substantial proportion of daily caloric intake toward later hours of the day [22]. Likewise, diet-induced thermogenesis appears substantially higher after breakfast than after dinner, indicating that identical caloric loads may be metabolically handled differently depending on the timing of intake [18,29]. From a circadian perspective, these findings may reflect reduced metabolic flexibility during the biological evening, when insulin sensitivity, glucose tolerance, and lipid oxidation progressively decline [18].

Consequently, concentrating the principal caloric load later in the active phase may favor positive energy balance and adipose tissue storage even without increased eating frequency. Together, these findings reinforce the concept that “when” calories are consumed may be partially dissociable from “how many” meals are consumed and support the growing evidence that meal timing and circadian alignment may influence metabolic regulation independently of meal frequency alone [14]. Similarly, emerging evidence regarding early time-restricted eating suggests that aligning food intake with earlier circadian phases may improve metabolic regulation and insulin sensitivity independently of total caloric intake, further supporting the biological relevance of meal timing in weight-related outcomes [30].

The strengthening of the association between dinner predominance and BMI trajectory after adjustment for baseline BMI is particularly relevant. Baseline BMI was inversely associated with subsequent BMI change, indicating that initial adiposity strongly influenced BMI trajectory during follow-up. After adjustment, dinner as the main meal remained significantly associated with both continuous BMI change and clinically significant weight gain, suggesting that this chrononutrition signal was not merely explained by baseline body size. This finding supports the robustness of the association and reinforces the importance of considering baseline anthropometric status in prospective studies of weight change, consistent with previous longitudinal evidence [31]. Additional stratified and interaction analyses further suggested that the association between dinner predominance and BMI trajectory was stronger among participants with higher baseline adiposity. These findings may indicate that individuals with overweight or obesity are more metabolically vulnerable to late eating-weighted caloric distribution, consistent with emerging chrononutrition evidence linking circadian eating misalignment with obesity-related metabolic susceptibility [32].

The broader clinical relevance of nutritional-behavior patterns is additionally supported by recent evidence showing that dietary interventions capable of improving body composition may also contribute to better health-related quality of life and metabolic outcomes [33]. Nevertheless, the exploratory nature of these analyses warrants cautious interpretation.

The increase in model explanatory performance after inclusion of baseline BMI also suggests that part of the variability in BMI trajectory during university transition is strongly influenced by pre-existing anthropometric status. The overall explanatory capacity of the primary multivariable model remained modest, suggesting that BMI trajectory during the transition to university life is influenced by numerous additional behavioral, psychosocial, environmental, circadian, and metabolic factors not comprehensively captured in the present study. This interpretation is consistent with contemporary obesity research indicating that differences in BMI and obesity-related outcomes are only partially explained by individual lifestyle behaviors, with substantial variability arising from other interacting determinants. Therefore, the statistically significant associations identified in this analysis should be interpreted cautiously and not as strong determinants of BMI change [34]. Although statistically significant, the observed effect sizes and the low proportion of explained variance suggest limited practical predictive utility when these chrononutrition-related variables are considered in isolation. By contrast, absolute late eating was not independently associated with BMI trajectory in our cohort. Although this may appear inconsistent with studies reporting adverse metabolic effects of delayed eating, it may reflect the distinction between clock time of food intake and the circadian distribution of daily caloric load. Recent evidence suggests that meal timing relative to the internal biological clock may be more metabolically relevant than clock time alone [17].

Therefore, a student who reports late eating but consumes only a light evening meal may not have the same metabolic exposure as a student whose largest daily meal occurs later in the day. This may explain why “dinner as main meal” was an independent positive correlate, whereas late eating defined as last caloric intake after 21:00 was not. Additionally, the relatively small proportion of participants consuming meals after 22:00 may have limited the ability to detect stronger dose–response relationships for extreme late eating. It is also possible that late eating behaviors act synergistically with other unmeasured circadian factors, including short sleep duration, social jetlag, late chronotype, or nocturnal snacking. This finding contrasts with much of the existing epidemiological and meta-analytic literature, which generally associates breakfast skipping with increased risk of overweight, obesity, and adverse metabolic outcomes [35,36].

However, the magnitude of the effect in our study was small, it did not correlate independently with clinically significant weight gain, and it attenuated after adjustment for baseline BMI. These findings suggest that breakfast skipping in this cohort may have reflected unmeasured behavioral factors, such as intentional caloric restriction, time pressure, financial constraints, stress-related appetite suppression, or prior weight-control attempts, rather than a causal protective effect. Reverse causality should also be considered, because participants attempting weight reduction or engaging in intentional caloric restriction may have been more likely to skip breakfast. In addition, residual confounding by unmeasured factors such as physical activity, sleep characteristics, psychological stress, diet quality, or socioeconomic conditions may have contributed to the observed association. The observed reduction in BMI among breakfast skippers should not automatically be interpreted as beneficial from a metabolic or nutritional perspective. Lower BMI may coexist with poorer diet quality, irregular eating behaviors, psychological stress, inadequate nutrient intake, or unfavorable body-composition changes that are not captured by BMI alone. Moreover, recent evidence suggests that breakfast skipping may be associated with adverse cardiometabolic outcomes, including metabolic syndrome and several of its components, highlighting that BMI reduction does not necessarily equate to improved overall metabolic health [37].

Studies in university populations have shown that breakfast skipping is frequently associated with irregular academic schedules, perceived stress, time constraints, poorer diet quality, delayed sleep schedules, and broader circadian-related lifestyle disruption [38,39]. Therefore, breakfast omission in young adults may not necessarily represent the same behavioral or metabolic construct observed in older adult populations. This interpretation is also supported by randomized and meta-analytic evidence indicating that eating versus skipping breakfast may not exert a uniform effect on obesity-related anthropometric outcomes [40]. Accordingly, the present findings should not be interpreted as supporting breakfast omission, but rather as highlighting the complex behavioral context surrounding breakfast habits in young adults. The attenuation of the association after adjustment for baseline BMI further supports the possibility of reverse causality or weight-control behaviors.

The absence of an independent association between meal frequency and BMI change is also noteworthy. Students reporting 2–3 meals/day and those reporting 4–5 meals/day showed nearly identical BMI trajectories, with negligible effect sizes. This supports the growing chrononutrition literature emphasizing meal timing, caloric distribution, eating window, and circadian alignment rather than meal count alone [14,32]. For university-based interventions, these findings suggest that discouraging late eating patterns and shifting the largest meal toward later hours of the day may be more relevant than recommending a specific number of daily meals.

Sex-stratified analyses further strengthened the interpretation of the main findings. Dinner as the principal meal was associated with BMI gain in both women and men, and interaction testing did not support meaningful sex-specific modification. This is important because sex differences were observed in baseline BMI and some chrononutrition behaviors, yet the dinner-related association remained directionally consistent across strata. Systematic evidence indicates that those who have late is consistently associated with late eating, poorer dietary quality, increased social jetlag, and greater obesity risk, particularly among young adults and university populations characterized by irregular behavioral schedules [41]. Although chronotype was not directly assessed in the present study, the consistency of the dinner-as-main-meal association across sexes suggests that late eating-related caloric predominance may represent a broadly relevant behavioral marker in this population.

The absence of chronotype and sleep assessment remains important because late chronotype is frequently associated with late eating, breakfast skipping, poorer diet quality, and higher BMI [42]. Sleep duration and sleep quality may also influence appetite regulation, physical activity, food choices, and weight-related outcomes in students. Recent longitudinal evidence in college populations has highlighted sleep quality as a potentially important factor in obesity-related changes [43]. Future studies should therefore integrate chronotype, sleep duration, sleep regularity, social jetlag, and objective or app-based meal-timing assessments to better characterize the circadian context of university eating behaviors. The integration of wearable-device monitoring, ecological momentary assessment, and smartphone-based dietary logging may further improve the precision of chrononutrition research and reduce reliance on retrospective self-report measures.

The practical implications of these findings extend to university-based lifestyle and nutritional interventions targeting metabolic health in young adults. Rather than emphasizing restrictive dietary messages, university health programs could promote earlier caloric distribution by encouraging students to make lunch, rather than dinner, the principal meal of the day. This recommendation is biologically plausible, behaviorally simple, and compatible with campus-level strategies such as protected lunch breaks, healthier midday canteen options, reducing late eating behaviors, and educational initiatives focused on chrononutrition and circadian eating behaviors during first-year orientation. Physical-activity-based interventions in university settings have also been identified as feasible approaches for improving weight-related and metabolic outcomes [44]. Moreover, growing evidence suggests that dietary patterns and meal-related behaviors exert broad effects extending beyond obesity alone, influencing gastrointestinal physiology, metabolic regulation, inflammatory pathways, and long-term clinical outcomes [45]. Chrononutrition-informed interventions may therefore represent a promising and more acceptable strategy for students because they focus on meal organization and circadian alignment rather than caloric deprivation alone.

Methodological aspects strengthen the interpretation of the present findings, including the prospective design, large study population, standardized anthropometric assessment, and simultaneous evaluation of multiple chrononutrition-related behaviors within the same analytical framework. The use of both continuous and clinically meaningful BMI outcomes, together with extensive sensitivity analyses, improved the robustness of the statistical interpretation. Additionally, baseline BMI-adjusted models, sex-stratified analyses, interaction testing, and effect-size estimation allowed a more nuanced evaluation of the observed associations. The simultaneous assessment of meal timing, meal distribution, and meal frequency also provided a broader characterization of chrononutrition-related behaviors than approaches focused exclusively on isolated eating variables.

Several limitations should also be considered. Chrononutrition-related behaviors were assessed only at follow-up (T1), limiting the ability to establish temporal ordering between eating behaviors and BMI change. Because chrononutrition-related behaviors were measured only at T1, temporal directionality between eating behaviors and BMI trajectory cannot be definitively established. Consequently, the identified associations should be interpreted as concurrent behavioral correlates rather than temporally established determinants of BMI change, and reverse causality cannot be excluded. Furthermore, the questionnaire focused on selected chrononutrition-related behaviors and meal-timing patterns relevant to the objectives of the present study rather than on a comprehensive assessment of chrononutrition and lifestyle factors. As such, formal psychometric validation procedures were not performed, and the questionnaire was not intended to serve as a validated chrononutrition instrument. Consequently, several potentially important determinants of BMI trajectory and metabolic health were not evaluated in the present study, including total energy intake, macronutrient composition, dietary quality, alcohol consumption, snacking behaviors, ultra-processed food intake, chronotype, sleep duration and quality, social jetlag, stress-related factors, physical activity, and socioeconomic characteristics. The absence of these variables increases the possibility of residual confounding and limits the ability to disentangle the independent contribution of chrononutrition-related behaviors from broader lifestyle and behavioral factors. Additionally, the “main meal of the day” variable reflected subjective participant perception and was not standardized according to caloric intake, portion size, or objective nutritional composition, which may have introduced interpretative variability across participants. Because chrononutrition exposures were self-reported, recall bias and behavioral misclassification cannot be excluded completely. Similarly, BMI calculations were based on self-reported height and weight rather than direct anthropometric measurements. Self-reported anthropometric measures may underestimate body weight and overestimate height, potentially influencing BMI classification and the precision of longitudinal BMI-change estimates. Although the same self-report methodology was applied consistently at both assessment points, measurement error and reporting bias cannot be fully excluded. Residual confounding related to socioeconomic status, residence type, academic workload, or emotional eating patterns may likewise have influenced the observed associations. Additionally, because multiple univariate, multivariable, stratified, and interaction analyses were conducted, the possibility of type I error inflation cannot be completely excluded, particularly for secondary and exploratory findings. Furthermore, the study was conducted within a single university setting, which may limit generalizability to other student populations or sociocultural environments. Although approximately one-third of the eligible student population participated in the study, voluntary participation may have introduced selection bias, and the possibility that respondents differed from non-participants in health-related behaviors cannot be excluded. Finally, BMI does not differentiate fat mass from lean mass, and future studies should incorporate additional anthropometric and metabolic measures, including waist circumference, body-composition analysis, and metabolic biomarkers, to provide a more comprehensive characterization of metabolic health.

## 5. Conclusions

This prospective study suggests that during the transition to university life, the timing and distribution of caloric intake may be associated with BMI trajectory more strongly than meal frequency alone. Among the evaluated chrononutrition-related variables, self-perceived dinner predominance emerged as the chrononutrition-related behavior most consistently associated with BMI trajectory and clinically significant weight gain, whereas meal frequency and isolated late eating were not independently associated with BMI trajectory after multivariable adjustment.

The inverse association observed between breakfast skipping and BMI change should be interpreted cautiously, particularly because it contrasts with much of the existing literature and may reflect reverse causality, intentional weight-control behaviors, stress-related appetite suppression, or residual confounding rather than a protective metabolic effect.

These findings support the possibility that chrononutrition-related behaviors may be associated with weight regulation and BMI trajectory independently of traditional dietary metrics focused primarily on meal quantity or frequency. However, the explanatory capacity of the primary multivariable model was limited, indicating that these variables accounted for only a small proportion of the variability in BMI change and should not be interpreted as strong predictors of weight-related outcomes. However, because chrononutrition-related behaviors were assessed only at follow-up, the observed associations should not be interpreted as evidence of causal relationships.

The observed association between dinner predominance and less favorable BMI trajectories suggests that the temporal distribution of caloric intake may represent an important behavioral marker of weight-related outcomes during emerging adulthood. However, because chrononutrition-related behaviors were assessed only at follow-up, temporal directionality cannot be established and these findings should be interpreted as concurrent associations rather than evidence of causation. Future longitudinal studies incorporating repeated chrononutrition assessments, comprehensive dietary characterization, circadian measures, physical activity, and metabolic biomarkers are needed to better clarify the temporal and mechanistic relationships between chrononutrition behaviors and weight regulation during young adulthood.

## Figures and Tables

**Table 1:** Baseline characteristics of the study population according to sex (*N* = 921).

Variable	Overall (*N* = 921)	Women (*n* = 558)	Men (*n* = 363)	*p*-Value ^a^
Age stratum, *n* (%)				
18–20 years	443 (48.1)	269 (48.2)	174 (47.9)	0.989
21–24 years	478 (51.9)	289 (51.8)	189 (52.1)	
BMI_previous (kg/m^2^)				
Mean ± SD	23.72 ± 3.95	22.70 ± 3.80	25.30 ± 3.64	<0.001
Median (IQR)	23.49 (21.02–26.00)	22.34 (20.20–24.61)	25.07 (22.81–27.50)	
BMI_actual (kg/m^2^)				
Mean ± SD	23.27 ± 3.84	22.29 ± 3.68	24.78 ± 3.61	<0.001
Median (IQR)	22.98 (20.49–24.91)	22.07 (19.92–23.99)	24.34 (22.40–26.62)	
Diff_BMI (kg/m^2^)				
Mean ± SD	−0.45 ± 1.07	−0.41 ± 1.15	−0.52 ± 0.91	0.103
Median (IQR)	−0.34 (−1.19 to 0.19)	−0.21 (−1.22 to 0.24)	−0.56 (−1.15 to 0.13)	
Meals/day, *n* (%)				
2–3 meals/day	778 (84.5)	471 (84.4)	307 (84.6)	1.000
4–5 meals/day	143 (15.5)	87 (15.6)	56 (15.4)	
Main meal of the day, *n* (%)				
Breakfast	76 (8.3)	49 (8.8)	27 (7.4)	<0.001
Lunch	537 (58.3)	351 (62.9)	186 (51.2)	
Dinner	308 (33.4)	158 (28.3)	150 (41.3)	
Daily breakfast consumption = Yes, n (%)	274 (29.8)	167 (29.9)	107 (29.5)	0.824
Breakfast including a main dish = Yes, n (%)	539 (58.5)	318 (57.0)	221 (60.9)	0.174
Late eating categories, *n* (%)				
Before 18:00	171 (18.6)	117 (21.0)	54 (14.9)	0.032
19:00–20:00	366 (39.7)	221 (39.6)	145 (39.9)	
21:00–22:00	335 (36.4)	192 (34.4)	143 (39.4)	
After 22:00	49 (5.3)	28 (5.0)	21 (5.8)	

^a^ *p*-values represent comparisons between women and men. Welch independent-samples *t*-test was used for continuous variables and chi-square (χ^2^) test for categorical variables. Continuous variables are presented as mean ± standard deviation (SD) and median with interquartile range (IQR). Percentages may not total exactly 100% because of rounding. BMI, body mass index; Diff_BMI, BMI_actual − BMI_previous; SD, standard deviation; IQR, interquartile range.

**Table 2:** Patterns of BMI change during the first university year *(N* = 921).

Pattern of BMI Change	Definition	*n* (%)	Diff_BMI, Mean ± SD (kg/m^2^)	Diff_BMI, Median (IQR)
BMI decrease	Diff_BMI < −0.5 kg/m^2^	444 (48.2)	−1.36 ± 0.74	−1.16 (−1.69, −0.78)
Stable BMI	−0.5 ≤ Diff_BMI ≤ +0.5 kg/m^2^	356 (38.7)	−0.08 ± 0.29	−0.10 (−0.31, 0.16)
BMI increase	Diff_BMI > +0.5 kg/m^2^	121 (13.1)	+1.35 ± 0.85	+1.06 (+0.72, +1.65)
Clinically significant weight gain ^a^	Diff_BMI > +1.0 kg/m^2^	66 (7.2)	+1.79 ± 0.79	+1.51 (+1.19, +2.14)

^a^ Clinically significant weight gain represents a subset of participants included within the “BMI increase” category and was retained as a secondary outcome for logistic regression analyses. Continuous variables are presented as mean ± standard deviation (SD) and median with interquartile range (IQR). BMI, body mass index; Diff_BMI, BMI_actual − BMI_previous; SD, standard deviation; IQR, interquartile range.

**Table 3:** Diff_BMI according to daily meal frequency.

Daily Meal Frequency	*n* (%)	Diff_BMI, Mean ± SD (kg/m^2^)	Diff_BMI, Median (IQR)	*p*-Value ^a^	*p*-Value ^b^	Cohen’s D (95% CI)
2–3 meals/day	778 (84.5)	−0.46 ± 1.08	−0.39 (−1.21 to 0.18)	0.624	0.402	−0.045 (−0.223 to 0.134)
4–5 meals/day	143 (15.5)	−0.41 ± 1.00	−0.18 (−1.16 to 0.23)			reference group

^a^ Welch independent-samples *t*-test. ^b^ Mann–Whitney U test. Continuous variables are presented as mean ± standard deviation (SD) and median with interquartile range (IQR). Diff_BMI, BMI_actual − BMI_previous; SD, standard deviation; IQR, interquartile range.

**Table 4:** Comparison of BMI outcomes according to habitual breakfast consumption.

Outcome	Daily Breakfast Consumption = Yes (*n* = 274)	Breakfast Skipping = Yes (*n* = 647)	Mean Difference/OR (95% CI) ^c^	*p*-Value ^a^	*p*-Value ^b^	Cohen’s D (95% CI)
Diff_BMI (kg/m^2^)						
Mean ± SD	−0.32 ± 0.98	−0.50 ± 1.10	−0.18 (−0.33 to −0.04)	0.013	0.007	0.17 (0.03 to 0.31)
Median (IQR)	−0.16 (−0.96 to 0.31)	−0.50 (−1.27 to 0.14)	-	-	-	-
BMI_actual (kg/m^2^)						
Mean ± SD	23.33 ± 4.01	23.25 ± 3.78	−0.08 (−0.64 to 0.47)	0.767	-	0.02 (−0.12 to 0.16)
Clinically significant weight gain (Diff_BMI > +1 kg/m^2^), n (%)	22 (8.0)	44 (6.8)	OR 0.72 (0.43–1.22)	0.224 ^d^	-	-

^a^ Welch independent-samples *t*-test. ^b^ Mann–Whitney U test. ^c^ Mean difference calculated relative to the daily breakfast consumption reference group; odds ratios (ORs) are reported for breakfast skipping versus daily breakfast consumption. ^d^ Univariable logistic regression analysis. Continuous variables are presented as mean ± standard deviation (SD) and median with interquartile range (IQR). Categorical variables are presented as absolute frequencies and percentages. Diff_BMI, BMI_actual − BMI_previous; BMI, body mass index; SD, standard deviation; IQR, interquartile range; OR, odds ratio; CI, confidence interval.

**Table 5:** Diff_BMI according to late eating categories of the day.

Late Eating Categories	*n* (%)	Diff_BMI, Mean ± SD (kg/m^2^)	Diff_BMI, Median (IQR)	Linear Regression β (95% CI) ^a^	*p*-Value (Unadjusted)	ANCOVA β (95% CI) ^b^	*p*-Value (Adjusted)
Before 18:00 (reference)	171 (18.6)	−0.41 ± 0.93	−0.20 (−1.14 to 0.17)	-	-	-	-
19:00–20:00	366 (39.7)	−0.50 ± 1.12	−0.55 (−1.22 to 0.17)	−0.09 (−0.29 to 0.10)	0.344	−0.11 (−0.30 to 0.09)	0.272
21:00–22:00	335 (36.4)	−0.43 ± 1.05	−0.23 (−1.24 to 0.23)	−0.02 (−0.22 to 0.17)	0.816	−0.03 (−0.23 to 0.16)	0.737
After 22:00	49 (5.3)	−0.34 ± 1.20	−0.36 (−1.06 to 0.42)	+0.07 (−0.27 to 0.41)	0.694	+0.07 (−0.27 to 0.41)	0.667

Overall ANOVA F(3, 917) = 0.58, *p* = 0.627; Kruskal–Wallis H = 2.31, *p* = 0.510 ^a^ Linear regression analysis of Diff_BMI according to late eating category, using “Before 18:00” as the reference category. ^b^ ANCOVA additionally adjusted for sex (male vs. female) and age stratum (21–24 vs. 18–20 years). Continuous variables are presented as mean ± standard deviation (SD) and median with interquartile range (IQR). Diff_BMI, BMI_actual − BMI_previous; ANCOVA, analysis of covariance; SD, standard deviation; IQR, interquartile range; CI, confidence interval.

**Table 6:** Multiple linear regression analysis for Diff_BMI and multivariable logistic regression analysis for clinically significant weight gain (Diff_BMI > +1 kg/m^2^) (*N* = 921).

Variables	Linear Regression β (kg/m^2^)	95% CI	*p*-Value ^a^	Adjusted OR (aOR)	95% CI	*p*-Value ^b^	Standardized β
Intercept	−0.28	−0.46 to −0.09	0.003	0.13	0.07 to 0.25	<0.001	—
Daily meal frequency = 4–5 meals/day (reference: 2–3 meals/day)	0.005	−0.19 to 0.19	0.961	0.88	0.43 to 1.78	0.719	0.002
Breakfast skipping (reference: daily breakfast consumption)	−0.19	−0.35 to −0.04	0.013	0.67	0.39 to 1.15	0.145	−0.083
Late eating (≥21:00)	0.02	−0.12 to 0.16	0.807	1.10	0.66 to 1.84	0.707	0.008
Main meal = breakfast (reference: lunch)	−0.24	−0.51 to 0.02	0.067	0.29	0.07 to 1.24	0.094	−0.063
Main meal = dinner (reference: lunch)	0.22	0.07 to 0.37	0.004	1.43	0.84 to 2.43	0.191	0.099
Sex = male (reference: female)	−0.15	−0.29 to −0.01	0.040	0.36	0.19 to 0.66	<0.001	−0.068
Age = 21–24 years (reference: 18–20 years)	−0.07	−0.21 to 0.06	0.289	0.94	0.56 to 1.56	0.796	−0.035

Multiple linear regression: F(7, 913) = 3.47, model *p* = 0.001; R^2^ = 0.026; adjusted R^2^ = 0.018. Multivariable logistic regression: log-likelihood ratio *p* = 0.006; McFadden pseudo R^2^ = 0.042. ^a^ Multiple linear regression model with Diff_BMI as the dependent variable. ^b^ Multivariable logistic regression model with clinically significant weight gain (Diff_BMI > +1 kg/m^2^) as the dependent variable. Statistically significant associations (*p* < 0.05) are highlighted in bold. Diff_BMI, BMI_actual − BMI_previous; β, regression coefficient; aOR, adjusted odds ratio; CI, confidence interval; OR, odds ratio.

**Table 7:** Baseline-BMI-adjusted multivariable regression models for Diff_BMI and clinically significant weight gain (Diff_BMI > +1 kg/m^2^) (*N* = 921).

Variables	β (kg/m^2^)	Standardized β	95% CI	*p*-Value (Linear)	aOR	95% CI	*p*-Value (Logistic)
Intercept	2.00	-	1.54 to 2.45	<0.001	8.88	1.55 to 50.87	0.014
BMI_previous (per +1 kg/m^2^)	−0.111	−0.46	−0.131 to −0.090	<0.001	0.81	0.75 to 0.88	<0.001
Daily meal frequency = 4–5 meals/day (reference: 2–3 meals/day)	−0.02	−0.01	−0.20 to 0.16	0.803	0.82	0.40 to 1.70	0.599
Breakfast skipping (reference: daily breakfast consumption)	−0.13	−0.05	−0.28 to 0.01	0.068	0.74	0.43 to 1.29	0.290
Late eating (≥21:00)	0.02	0.01	−0.11 to 0.15	0.773	1.08	0.64 to 1.83	0.780
Main meal = breakfast (reference: lunch)	−0.22	−0.06	−0.47 to 0.02	0.075	0.28	0.06 to 1.22	0.089
Main meal = dinner (reference: lunch)	0.67	0.24	0.51 to 0.83	<0.001	2.91	1.61 to 5.27	<0.001
Sex = male (reference: female)	0.10	0.03	−0.04 to 0.24	0.154	0.56	0.29 to 1.07	0.077
Age = 21–24 years (reference: 18–20 years)	0.06	0.02	−0.07 to 0.19	0.372	1.15	0.68 to 1.94	0.601

Multiple linear regression: F(8, 912) = 17.54, model *p* < 0.001; R^2^ = 0.133; adjusted R^2^ = 0.126. Multivariable logistic regression: log-likelihood ratio *p* < 0.001; McFadden pseudo R^2^ = 0.098. β, regression coefficient; aOR, adjusted odds ratio; CI, confidence interval.

**Table 8:** Stratified multiple linear regression analyses for Diff_BMI according to baseline BMI category.

Variables	Women (*n* = 558)			Men (*n* = 363)		
	β (kg/m^2^)	95% CI	*p*-Value	β (kg/m^2^)	95% CI	*p*-Value
Intercept	−0.32	−0.54 to −0.09	0.006	−1.08	−1.44 to −0.73	<0.001
Meals/Day = 4–5 (vs. 2–3)	0.05	−0.19 to 0.30	0.674	−0.09	−0.35 to 0.17	0.498
Breakfast skipping (No vs. Yes)	−0.07	−0.27 to 0.12	0.473	−0.32	−0.54 to −0.10	0.004
Late eating (≥21:00)	0.11	−0.07 to 0.29	0.243	−0.07	−0.26 to 0.13	0.506
Main meal = breakfast (vs. lunch)	−0.23	−0.52 to 0.07	0.135	−0.05	−0.58 to 0.47	0.841
Main meal = dinner (vs. lunch)	0.37	0.13 to 0.62	0.003	0.77	0.54 to 0.99	<0.001
Age = 21–24 (vs. 18–20)	−0.10	−0.28 to 0.07	0.237	0.16	−0.05 to 0.37	0.136
Sex = male (vs. female)	0.04	−0.15 to 0.24	0.657	−0.13 to 0.29	−0.13 to 0.29	0.435

Models adjusted for all variables listed. β, regression coefficient; CI, confidence interval. Statistically significant associations (*p* < 0.05) are shown in bold. The effect of dinner as the main meal on BMI gain was substantially stronger among participants who were overweight or obese at baseline.

**Table 9:** Sex-stratified multivariable linear regression analyses for Diff_BMI.

Variables	Women (*n* = 558)			Men (*n* = 363)		
	β (kg/m^2^)	95% CI	*p*-Value	β (kg/m^2^)	95% CI	*p*-Value
Intercept	−0.23	−0.48 to 0.01	0.062	−0.51	−0.75 to −0.26	<0.001
Meals/Day = 4–5 (vs. 2–3)	0.02	−0.25 to 0.28	0.887	0.002	−0.26 to 0.26	0.990
Breakfast skipping (No vs. Yes)	−0.19	−0.41 to 0.02	0.077	−0.19	−0.40 to 0.02	0.072
Late eating (≥21:00)	−0.01	−0.20 to 0.19	0.943	0.07	−0.12 to 0.26	0.491
Main meal = breakfast (vs. lunch)	−0.31	−0.64 to 0.02	0.062	−0.03	−0.49 to 0.43	0.900
Main meal = dinner (vs. lunch)	0.23	0.01 to 0.45	0.037	0.22	0.02 to 0.42	0.031
Age = 21–24 (vs. 18–20)	−0.14	−0.33 to 0.05	0.153	0.03	−0.16 to 0.22	0.781

Women: model F(6, 551) = 2.36, *p* = 0.030; adjusted R^2^ = 0.014. Men: model F(6, 356) = 1.74, *p* = 0.113; adjusted R^2^ = 0.012. β, regression coefficient; CI, confidence interval.

**Table 10:** Interaction analyses between sex and chrononutrition-related correlates for Diff_BMI.

Interaction Model	Interaction Term	β (kg/m^2^)	95% CI	*p*-Value	Main Effect
Sex × Dinner-as-main-meal	MainDinner × SexM	−0.019	−0.314 to 0.276	0.899	Dinner β = 0.231 (95% CI 0.033 to 0.428; *p* = 0.022)
Sex × Late eating	Late eating × SexM	0.060	−0.224 to 0.344	0.677	Late eating β = −0.007 (95% CI −0.186 to 0.173; *p* = 0.944)
Both interactions (joint model)	MainDinner × SexM	−0.027	−0.324 to 0.271	0.860	Dinner β = 0.233 (*p* = 0.021)
	Late eating × SexM	0.063	−0.223 to 0.349	0.664	Late eating β = −0.008 (*p* = 0.934)

Each interaction model is adjusted for meals/day, breakfast skipping, the remaining main-meal category, age stratum, and (where not interacting) sex. None of the multiplicative interaction terms reached statistical significance, indicating no evidence that sex modifies the association of dinner-as-main-meal or of late eating with Diff_BMI. β, regression coefficient; CI, confidence interval.

## Data Availability

The de-identified dataset supporting the conclusions of this article is available from the corresponding author upon reasonable request.

## Supplementary Materials

The following supporting information can be downloaded at: https://www.mdpi.com/article/10.3390/nu18121975/s1, Supplementary File S1: Questionnaire (English version).

## Abbreviations

The following abbreviations are used in this manuscript:

aOR	Adjusted odds ratio
ANCOVA	Analysis of covariance
ANOVA	Analysis of variance
BMI	Body mass index
BMI_actual	Body mass index assessed after one academic year
BMI_previous	Body mass index assessed at university entry
CI	Confidence interval
Diff_BMI	Difference between BMI_actual and BMI_previous
IQR	Interquartile range
OR	Odds ratio
SD	Standard deviation
T0	Baseline assessment at university entry
T1	Follow-up assessment after one academic year
VIF	Variance inflation factor
WHO	World Health Organization
χ^2^	Chi-square test
